# Efficacy of intravaginal electrical stimulation added to bladder training in women with idiopathic overactive bladder: A prospective randomized controlled trial

**DOI:** 10.1590/S1677-5538.IBJU.2021.0161

**Published:** 2021-08-01

**Authors:** Necmettin Yildiz, Hakan Alkan, Ayse Sarsan

**Affiliations:** 1 Pamukkale University Faculty of Medicine Department of Physical Medicine and Rehabilitation Denizli Turkey Department of Physical Medicine and Rehabilitation, Pamukkale University Faculty of Medicine, Denizli, Turkey

**Keywords:** Urinary Bladder, Overactive, Electric Stimulation Therapy, Controlled Clinical Trial [Publication Type]

## Abstract

**Purpose:**

To evaluate the efficacy of intravaginal electrical stimulation (IVES) added to bladder training (BT) on incontinence-related quality of life (QoL) and clinical parameters in women with idiopathic overactive bladder (OAB).

**Materials and Methods:**

Sixty-two women with idiopathic OAB were randomized into two groups using the random numbers generator as follows: Group 1 received BT alone (n:31), and Group 2 received BT+IVES (n:31). IVES was performed for twenty minutes three days a week over a course of eight weeks for a total of 24 sessions. Patients were evaluated in terms of incontinence severity (24-hour pad test), pelvic floor muscles strength (perineometer), 3-day voiding diary (frequency of voiding, nocturia, incontinence episodes and number of pads), symptom severity (OAB-V8), incontinence-related QoL (IIQ-7), treatment success (positive response rate), cure/improvement rate and treatment satisfaction (Likert scale).

**Results:**

A statistically significant improvement was found in all parameters for all groups at the end of the treatment compared to the baseline values except pelvic floor muscles strength in Group 1 (p <0.05). At the end of treatment, incontinence severity, frequency of voiding, nocturia, incontinence episodes, number of pads, symptom severity, and QoL were significantly improved in Group 2 compared to Group 1 (p <0.05). Treatment satisfaction, cure/improvement, and positive response rates were significantly higher in group 2 compared to Group 1 (p <0.05).

**Conclusion:**

We conclude that BT+IVES were more effective than BT alone on both incontinence-related QoL and clinical parameters in women with idiopathic OAB.

## INTRODUCTION

Overactive bladder (OAB) is defined as urinary urgency, usually accompanied by frequency and nocturia, with or without urgency urinary incontinence (UUI) according to the International Continence Society (1). Many drugs such as oral anti-muscarinic agents and oral ß3 adrenoreceptor agonist (mirabegron) and first-line conservative therapeutic options are commonly used for UUI and OAB, including electrical stimulation (ES), pelvic floor muscle (PFM) training and behavioral therapies such as lifestyle changes and bladder training (BT) (1-6). BT involves a systematic voiding regimen to lengthen the interval between voids until an acceptable pattern has been restored (2, 4, 5). BT is effective for the improvement of urinary incontinence in women and it's recommended as first-line therapy for adults with UUI (strong recommendation) (2). ES is one of the techniques used in urogynecological rehabilitation. Depending on how electrodes are applied, a differentiation is made between transcutaneous ES (via suprapubic attachment of electrodes, intra-vaginal/anal plug electrodes, etc.) and percutaneous ES (of the tibial nerve, electroacupuncture, etc.) (6). Intravaginal ES (IVES) is a conservative treatment option, described more than 40 years ago. IVES is used in patients with OAB and UUI, for detrusor inhibition. According to the European Association Urology Guidelines, in adults with urinary incontinence, ES may improve urinary incontinence compared to sham treatment (2). Despite that, there is controversy in scientific literature regarding its effectiveness as monotherapy (7).

In urogynecological rehabilitation, BT and IVES are frequently used together in the treatment of women with idiopathic OAB. While BT is a therapeutic option in which the patient is active during the treatment process, the patient is passive during the IVES application. BT and IVES are effective in completely different ways in women with idiopathic OAB (1-5). A combination of BT and IVES may have an additive effect in women with OAB. However, conservative treatment combinations such as BT and IVES are not yet recommended in the guidelines. Up to our knowledge, there are only two studies including BT+IVES treatment arm in women with idiopathic OAB in the literature. The results of these two studies are contradictory (8, 9). In the light of our clinical experience, we think that this issue is still open for research. Therefore, this study aimed to evaluate the efficacy of IVES added to BT on incontinence-related quality of life (QoL) and clinical parameters in women with idiopathic OAB.

## MATERIAL AND METHODS

This study was a prospective, randomized controlled trial. The trial was conducted at the Urogynecological Rehabilitation Unit of University Hospital, Physical Medicine and Rehabilitation Department between May 2020 and January 2021. This study was approved by the Institutional Review Board of our University (60116787-020/29687) and it was registered with ClinicalTrials.gov number, NCT04389307. All women signed consent forms before participation.

We calculated the sample size using the reduction of urge incontinence episodes after two modalities of treatments (ES and Sham ES) in patients with OAB. As previously published, ES treatment succeeded to reduce incontinence episodes (positive response rate) in 81.3% compared to 32.1% after Sham ES (p=0.001) (9). The optimum sample size should be 28 cases in each arm with a level of significance of 95% (α=5%), a power of 95% (ß=0.05) when an expected 50% or greater improvement of incontinence episodes reported in the previous study (10). Taking possible withdrawals (10% of the number of patients) into account, 62 women (31 women for each group) were enrolled. Sample size calculation was done using G* Power 3.1 Statistical Power Analysis for Microsoft Windows and Mac. Statistics were performed by another physician who was blinded to groups.

We recruited 81 women with complaints about OAB who were referred to the Urogynecological Rehabilitation Unit and other related outpatient clinics. Women over the age of 18 with the clinical diagnosis of idiopathic OAB, who had urodynamically confirmed detrusor overactivity (the presence of detrusor contractions in the filling phase of saline cystometry) and who were intolerant or unresponsive to antimuscarinics and discontinued at least 4 weeks ago, and who could be able to give written informed consent and understand the procedures, were included in this study. The criteria for exclusion were as follows: women who had stress urinary incontinence; a history of conservative therapy (BT, ES) for OAB within 6 months; urogynecological surgery within 3 months; current vulvovaginitis or urinary tract infections or malignancy; pregnancy; cardiac pacemaker or implanted defibrillator; anatomic structural disorders of the genital region that did not allow to apply the vaginal probe; strength of PFM less than 3/5 (graded as modified Oxford scale, min:0-max:5); pelvic organ prolapse quantification (POP-Q) (stage 2 or more); neurogenic bladder; peripheral or central neurologic pathology; ultrasonographic evidence of post-void residual urine volume more than 100mL (using Telemed Micrus portable ultrasonography (the Lithuania) device (11), and allergy to condom or lubricant gel that is used with perineometer/vaginal probe.

**Figure 1:**
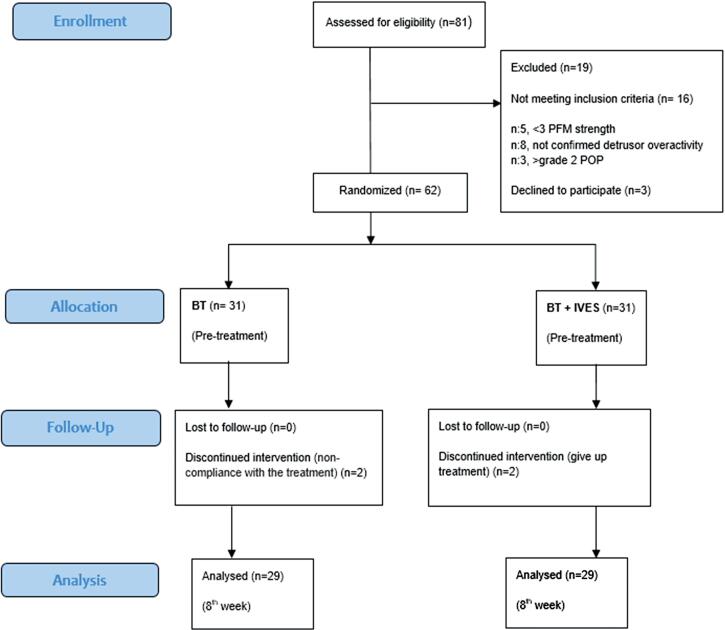
CONSORT participant flow diagram for randomized, controlled trials of nonpharmacologic treatment, BT, Bladder training; IVES, Intravaginal electrical stimulation; PFM, pelvic floor muscle; POP, pelvic organ prolapse.

Eighty-one women with idiopathic OAB were recruited for eligibility and sixty-two of them who fulfilled inclusion/exclusion criteria were included into this study. The flow chart is shown in Figure 1. Women were assigned to intervention groups by generating the random allocation sequence. By using a random number generator, 62 women were randomized into two groups as follows: Group 1 received BT alone (n:31), Group 2 received BT+IVES (n:31). A random allocation sequence was generated at 1:1 ratio.

### Group 1: Bladder Training (BT)-Control group

All women were informed about BT, consisting of four stages for 30 minutes. Then, it was given as a written brochure to be implemented as a home program. At the first stage, the women were familiarized with the location of the PFM and the pelvic anatomy and pathophysiology. After that information session, squeezing the PFM was shown in practice at least once to use in the urgency suppression strategies via digital palpation technique. In the second stage, including urgency suppression strategies, it was aimed to delay urination, to inhibit detrusor contraction, and to prevent urgency; by squeezing the PFM several times in a row, breathing deeply, giving their attention to another job for a while, and self-motivating. In the third stage, timed voiding program was started. It was carried out in 2 steps: a timed voiding and increasing the time between urination considering the voiding diary. At the last stage, the women were encouraged to continue BT (4, 5, 8, 9, 12).

### Group 2: Bladder Training+Intravaginal Electrical Stimulation (BT+IVES)

IVES was applied in addition to BT in this group. IVES was performed in lithotomy position via a stimulation device (Enraf Nonius Myomed 632) with a vaginal probe. IVES was performed three days a week, 20 minutes a day, a total of 24 sessions for 8 weeks. The stimulation parameters were frequency at 10Hz, a 5-10s work-rest cycle and a 100ms pulse width. The symmetric biphasic pulse wave could be delivered over a range of 1-100mA (according to the patient's discomfort level feedback) (9, 13, 14). IVES sessions were performed by an experienced urogynecologic rehabilitation nurse in Group 2.

During the treatment, all women were advised to continue the medical treatment which was not related to incontinence. Participants were asked to fill in a one-day bladder diary biweekly to continue the timed voiding program, which is part of BT in two groups. Compliance with the BT was achieved with the daily checklist during 8 weeks and the bladder diaries of women were checked biweekly to rearrange the timed voiding program. Women who did not fill more than 20% of the daily checklist for two groups and women who missed 10% of therapy sessions (more than 2sessions) for Group 2 were excluded from the study.

### Evaluation Parameters

The primary outcome measure was an improvement in incontinence episodes (positive response rate), according to literature (10, 15). To determine positive response rate, reduction in incontinence episodes was collected from the 3-day bladder diary. Women with ≥50% reduction in incontinence episodes were considered positive responders (9, 16). Furthermore, the severity of incontinence, PFM strength, symptom severity, frequency of voiding, nocturia, number of pads as well as QoL was a secondary outcome measure. The 24-hour pad test was carried out to evaluate the severity of incontinence (17). PFM strength was evaluated with Peritron 9300 device (18). Overactive Bladder Questionnaire (OAB-V8) was used to evaluate symptom severity in patients with OAB in the study. The OAB-V8 consists of 8 questions in which patients can be classified as symptom severity: none (0), very little (1), a little (2), quite a few (3), very (4), and too many (5). The total score ranges from 0-40 (19-21). The frequency of voiding, nocturia, and the number of pads used were collected from the 3-day bladder diary. The Quality of Life-Incontinence Impact Questionnaire (IIQ7) was used to assess specific QoL related to incontinence (21, 22). In addition, cure-improvement rates and treatment satisfaction were evaluated. Women evaluated the change of their urinary incontinence on a 5-point Likert scale (5, very satisfied; 1, very unsatisfied) (9, 23). In a 24-hour pad test, incontinence that was under 1.3gr was considered as a cure. The improvement was assessed in terms of 50% and more reduction in wet weight compared to baseline measurements in the 24-hour pad test (17). All the evaluation parameters were performed by another physician who was blinded to groups in the initial visit and repeated at the end of the treatment (8^th^ week).

#### Statistics

SPSS17.0 software (SPSS, Chicago, IL) was used for the statistical analysis. In each group, measurable parameters were tested with the Kolmogorov-Smirnov test for the evaluation of normal distribution. Because the distributions were not normal, non-parametric tests were used in the statistical evaluation. Mann-Whitney U-test and X^2^ test were used for inter-group comparisons. Wilcoxon test was used for intra-group comparison of parameters at different times. P <0.05 was accepted as statistically significant.

## RESULTS

Two women withdrew because of doing BT irregularly in Group 1 and two women withdrew because of giving up treatment in Group 2. The data of dropouts were excluded from the study (Figure 1).

Demographic data at the beginning are shown in Table-1. There were no statistically significant differences in the demographic data. Table-2 shows the comparisons of the assessment parameters at baseline and the end of the treatment (8^th^ week) for each group. Two groups were comparable for the severity of incontinence, PFM strength, frequency of voiding, incontinence episodes, nocturia, number of pads, symptom severity, and QoL parameters at baseline (p >0.05) (Table-2).

**Table 1:** Demographic data of women.

		Group 1 n=29	Group 2 n=29	P1	P2
Age (year) (mean±SD)		56.44±11.62	55.24±10.57	0.779	
Height (cm) (mean±SD)		160.79±4.34	159.20±6.01	0.407	
Weight (kg) (mean±SD)		73.68±9.11	75.20±11.92	0.409	
BMI (kg/m^2^) (mean±SD)		28.19±3.93	29.74±4.82	0.080	
Duration of incontinence (month) (mean±SD)		84.00±61.16	79.86±66.60	0.685	
**Education, n(%)**					
	Primary	12(41.4)	22(75.9)		
	High school	8(27.6)	4(13.8)		
	>High school	9(31.0)	3(10.3)		0.064
**Smoking, n(%)**					
	No	24 (82.8)	26(89.7)		
	Yes	5(17.2)	3(10.3)		0.191
**Cup of tea/day, n(%)**					
	1-2 cup	12(41.4)	9(31.0)		
	≥3 cup	17(58.6)	20(69.0)		0.412
**Cup of coffee/day, n(%)**					
	No	12(41.4)	14(48.3)		
	1-2 cup	15(51.7)	14(48.3)		
	≥3 cup	2(6.9)	1(3.4)		0.768
**Alcohol intake, n(%)**					
	No	29(100)	28(96.6)		
	Yes	0(0)	1(3.4)		0.374
**Delivery, n(%)**					
	No	0(0)	1(3.4)		
	1-3	27(93.1)	20(69.0)		
	≥4	2(6.9)	8(27.6)		0.060
**Delivery type, n(%)**					
	No	0(0)	1(3.4)		
	NSVD	22(75.9)	26(89.7)		
	Sectio	7(24.1)	2(6.9)		0.097
**Episiotomy, n(%)**					
	No	16(55.2)	18(62.1)		
	Yes	13(44.8)	11(37.9)		0.594
**Menopausal status, n(%)**					
	Premenopause	13(44.8)	10(34.5)		
	Postmenopause	16(55.2)	19(65.5)		0.421
**HRT use, n(%)**					
	No	26(89.7)	28(96.6)		
	Yes	3(10.3)	1(3.4)		0.300

Group1, Bladder Training; Group2, Bladder Training + Intravaginal Electrical Stimulation; HRT, Hormon replacement therapy; BMI, Body mass index; NSVD, normal spontaneous vaginal delivery; P^1^, Mann-Whitney U-test; P^2^, Pearson X^2^ test.

**Table 2:** Comparison of treatment groups in assessment variables.

		Group 1 n=29	Group 2 n=29	Mann-Whitney-U test
**Severity of incontinence - 24-h Pad test (gr), (mean±SD)**				
Pretreatment		42.06±22.15	41.48±26.25	0.779
8^th^ week		26.65±20.69 *	7.05±11.97 *	0.001
**PFM strength - Perineometer (cmH2O), mean±SD**				
	Pretreatment	23.96±9.75	23.55±11.69	0.888
	8^th^ week	24.48±9.62	28.13±13.33 *	0.308
**Bladder diary**				
**a. Frequency, mean±SD**				
	Pretreatment	10.44±2.75	11.75±3.69	0.082
	8^th^ week	8.79±2.27 *	6.51±1.95 *	<0.001
**b. Nocturia, mean±SD**				
	Pretreatment	2.77±0.62	2.55±2.09	0.542
	8^th^ week	1.86±0.58 *	0.89±0.90 *	<0.001
**c. Incontinence episodes, mean±SD**				
	Pretreatment	4.00±1.82	3.82±2.76	0.178
	8^th^ week	2.68±1.83 *	0.68±1.10 *	<0.001
**d. Number of pads, mean±SD**				
	Pretreatment	3.55±2.38	3.31±2.17	0.602
	8^th^ week	2.51±1.80 *	1.58±1.63 *	0.017
**Symptom severity - OAB-V8, mean±SD**				
	Pretreatment	25.37±6.48	25.93±5.28	0.749
	8^th^ week	14.44±5.05 *	8.89±5.56 *	<0.001
**Quality of life - IIQ7, mean±SD**				
	Pretreatment	12.79±6.76	13.72±5.84	0.714
	8^th^ week	11.17±6.68 *	6.27±6.19 *	0.003
**Treatment satisfaction (****1**-**5****), mean±SD**				
	8^th^ week	2.97±0.38	4.41±0.73	<0.001

Group1, Bladder Training; Group2, Bladder Training + Intravaginal Electrical Stimulation; OAB-V8, Overactive Bladder Questionnaire; IIQ-7, Incontinence Impact Questionnaire; PFM, Pelvic floor muscle;

* P<0.05: Wilcoxon test comparison with baseline values

A statistically significant improvement was found in all parameters for two groups at the end of the treatment compared to the baseline values (p <0.05) except PFM strength in Group 1. Statistically significant high values were found in treatment success rate (positive response rate) which was determined as the primary outcome measure in Group 2 compared to Group 1 at the 8^th^ week (respectively, 86.2% and 41.4%, p <0.001) (Table-3). It was found that severity of incontinence, frequency of voiding, incontinence episodes, nocturia, number of pads, symptom severity, and QoL parameters were significantly improved in Group 2 at the 8^th^ week compared to Group 1 (p <0.05). Statistically higher treatment satisfaction scores were found in Group 2 compared to Group 1 (p <0.05). There were no statistically significant differences in PFM strength between the two groups. (Table-2). The cure/improvement rate was significantly higher in Group 2 compared to Group 1 at the 8^th^ week (Table-3).

**Table 3:** Intergroup comparison according to cure/improvement and positive response rates.

		Group 1 n:29	Group 2 n:29	P
**Treatment Success (Positive Response Rate), n (%)**				
	Yes	12 (41.4)	25 (86.2)	<0.001
	No	17 (58.6)	4 (13.8)	
**Improvement Rate, n (%)**				
	Improvement	14 (48.3)	26 (89.7)	
	No change	15 (51.7)	3 (10.3)	0.001
**Cure / Improvement Rate, n (%)**				
	Cure	6 (20.7)	13 (44.8)	
	Improvement	8 (27.6)	13 (44.8)	
	No change	15 (51.7)	3 (10.3)	0.003

Group1, Bladder Training; Group2, Bladder Training + Intravaginal Electrical Stimulation; P, Pearson X^2^ test.

No serious adverse events were reported in both groups except temporary discomfort due to vaginal irritation in four women in Group 2.

## DISCUSSION

In this prospective, randomized controlled trial, we have investigated the effectiveness of IVES added to BT on QoL and clinical parameters associated with incontinence in women with idiopathic OAB. As a result, we have observed a significant improvement in terms of severity of incontinence, frequency of voiding, incontinence episodes, nocturia, number of pads, symptom severity, and QoL at the 8th-week evaluations in two groups when compared with baseline. However, we found significant improvements in the severity of incontinence, frequency of voiding, incontinence episodes, nocturia, number of pads, symptom severity, and QoL, moreover higher treatment satisfaction, and better cure/improvement and positive response rates in the BT+IVES group than BT group at the end of the treatment. In general, IVES was well tolerated by women except for temporary discomfort due to vaginal irritation in four patients in the group including IVES in our study.

In the studies comparing the effectiveness of conservative treatment options in patients with idiopathic OAB, improvement rates in BT groups have been shown to range from 35-63% (4, 5, 9, 24). In our study, the positive response rate which was determined as the primary outcome measure in the BT group was found to be 41.4%, similar to other studies. Nevertheless, we think that non-standard BT programs and different evaluation parameters used in different studies are the main reason for different improvement rates.

Up to our knowledge, there are only two studies including the BT+IVES treatment arm (one of the four treatment arms in both) in women with idiopathic OAB in the literature (8, 9). In Berghmans et al. study (8), BT+IVES was not effective both from BT alone and from the untreated control group. While interpreting the results of this study, it should be taken into consideration that women received relatively few treatment sessions in that study in contrast to our study (respectively, once a week - 9 sessions and three times in a week - 24 sessions). However, in a recent study by Firinci et al. (9), BT+IVES was found to be more effective than BT alone. In our study, BT+IVES was observed to be more effective than BT alone in terms of incontinence-related QoL and all clinical parameters in accordance with the study of Firinci et al. (9), except the number of pads. In these two studies, the number of sessions (three times in a week - 24 sessions) was the same. However, there was no study comparing the frequency of stimulation such as daily, two or three times a week, and also weekly. Therefore, it should be kept in mind that different stimulation frequencies may lead to different results. We think that this issue is still open for research.

There was no study comparing the different electrical current parameters and thus, there is no evidence of which parameters are the most effective ones. The most commonly used frequency by the authors is 10Hz for UUI or OAB. Working and rest times range from 2sn to 10sn, the most commonly used being 5sn and 10sn, respectively (7, 9). All authors who described the intensity of electrical current used maximum intensity depending on the patient's tolerance (max 100mA). In most cases, the application time used was 20 minutes. The ES programs lasted between 4 weeks and 6 months, although generally IVES was applied for 8-12 weeks (7, 9). In our study, the most frequently used electrical current parameters, number of sessions, and application time were used in accordance with the literature (7, 9).

There are some limitations in our study. One of the limitations of this study is that there are no data about the long-term follow-up of the patients. Another limitation is that there are no data about urodynamics. We also assume that we ought not to ignore the effects of patients in our study results in the BT+IVES group's facility of having face-to-face interviews with health professionals in the hospital. In addition, a cost-effectiveness analysis was not performed in our study.

## CONCLUSION

We conclude that BT+IVES were more effective than BT alone on both clinical parameters and QoL associated with incontinence in women with idiopathic OAB. Our results may shed light on the potential for use of first-line conservative therapy combinations such as BT+IVES in clinical practice, but more studies are needed to evaluate this and long-term follow-ups are warranted.

## LIST OF ABBREVIATIONS

BT: Bladder Training
ES: Electrical Stimulation
IVES: Intravaginal Electrical Stimulation
OAB: Overactive Bladder
PFM: Pelvic Floor Muscle (PFM)
QoL: Quality of Life
UUI: Urgency Urinary Incontinence
